# Developing Medical Education Curriculum Reform Strategies to Address the Impact of Generative AI: Qualitative Study

**DOI:** 10.2196/53466

**Published:** 2023-11-30

**Authors:** Ikuo Shimizu, Hajime Kasai, Kiyoshi Shikino, Nobuyuki Araki, Zaiya Takahashi, Misaki Onodera, Yasuhiko Kimura, Tomoko Tsukamoto, Kazuyo Yamauchi, Mayumi Asahina, Shoichi Ito, Eiryo Kawakami

**Affiliations:** 1 Department of Medical Education Graduate School of Medicine Chiba University Chiba Japan; 2 Health Professional Development Center Chiba University Hospital Chiba Japan; 3 Department of Community-Oriented Medical Education Graduate School of Medicine Chiba University Chiba Japan; 4 Department of Artificial Intelligence Medicine Graduate School of Medicine Chiba University Chiba Japan

**Keywords:** artificial intelligence, curriculum reform, generative artificial intelligence, large language models, medical education, qualitative analysis, strengths-weaknesses-opportunities-threats (SWOT) framework

## Abstract

**Background:**

Generative artificial intelligence (GAI), represented by large language models, have the potential to transform health care and medical education. In particular, GAI’s impact on higher education has the potential to change students’ learning experience as well as faculty’s teaching. However, concerns have been raised about ethical consideration and decreased reliability of the existing examinations. Furthermore, in medical education, curriculum reform is required to adapt to the revolutionary changes brought about by the integration of GAI into medical practice and research.

**Objective:**

This study analyzes the impact of GAI on medical education curricula and explores strategies for adaptation.

**Methods:**

The study was conducted in the context of faculty development at a medical school in Japan. A workshop involving faculty and students was organized, and participants were divided into groups to address two research questions: (1) How does GAI affect undergraduate medical education curricula? and (2) How should medical school curricula be reformed to address the impact of GAI? The strength, weakness, opportunity, and threat (SWOT) framework was used, and cross-SWOT matrix analysis was used to devise strategies. Further, 4 researchers conducted content analysis on the data generated during the workshop discussions.

**Results:**

The data were collected from 8 groups comprising 55 participants. Further, 5 themes about the impact of GAI on medical education curricula emerged: improvement of teaching and learning, improved access to information, inhibition of existing learning processes, problems in GAI, and changes in physicians’ professionality. Positive impacts included enhanced teaching and learning efficiency and improved access to information, whereas negative impacts included concerns about reduced independent thinking and the adaptability of existing assessment methods. Further, GAI was perceived to change the nature of physicians’ expertise. Three themes emerged from the cross-SWOT analysis for curriculum reform: (1) learning about GAI, (2) learning with GAI, and (3) learning aside from GAI. Participants recommended incorporating GAI literacy, ethical considerations, and compliance into the curriculum. Learning with GAI involved improving learning efficiency, supporting information gathering and dissemination, and facilitating patient involvement. Learning aside from GAI emphasized maintaining GAI-free learning processes, fostering higher cognitive domains of learning, and introducing more communication exercises.

**Conclusions:**

This study highlights the profound impact of GAI on medical education curricula and provides insights into curriculum reform strategies. Participants recognized the need for GAI literacy, ethical education, and adaptive learning. Further, GAI was recognized as a tool that can enhance efficiency and involve patients in education. The study also suggests that medical education should focus on competencies that GAI hardly replaces, such as clinical experience and communication. Notably, involving both faculty and students in curriculum reform discussions fosters a sense of ownership and ensures broader perspectives are encompassed.

## Introduction

Artificial intelligence (AI) and its applications have great potential to resolve many challenges in health care, such as diagnostic implementation, diagnosis facilitation, and decision-making [1,2]. Furthermore, generative AI (GAI), represented by large language models (LLMs), can influence all activities in society because of its ability to perform a wide variety of natural language tasks, exhibiting deductive reasoning and chains of thought [3]. A typical example is ChatGPT, a representative generic LLM service recently developed by OpenAI [4]. Different from previous deep learning-based algorithms, LLMs can predict the likelihood of a given sequence of words based on the context of the words that come before it. Thus, LLMs can produce natural and meaningful language sequences by learning a sufficient amount of textual data.

As GAI algorithms are applied in a variety of domains, the potential and risks of GAI are being debated upon. In particular, the potential impact of GAI on education has become apparent. On the one hand, GAI has the potential to assist education in terms of providing an adaptive and personalized environment [5]. On the other hand, the impact of GAI on education is disputed [6], with studies raising concerns about the ethical considerations of ChatGPT [7], evaluation practices [8], scientific integrity [9], and potential negative effects on students’ higher order thinking skills [10]. As with past introductions of new technologies into education, GAI is disrupting traditional practices and forcing teachers to adapt to its potential positive and negative impacts [5]. For example, GAI is now capable of passing various certification examinations, including those for medical licensure in at least questions without images [11,12]. Thus, there is a movement in higher education to limit learners’ use of GAI. Further, the United Nations Educational, Scientific and Cultural Organization (UNESCO) has published the guidance for GAI in education and research [13], and the Ministry of Education, Culture, Sports, Science and Technology in Japan has also developed guidelines for the use of GAI in higher education in general [14]. These academic views on GAI do not uniformly declare that AI tools pose a serious threat to higher education. Although current GAI algorithms may have factual errors and biases, many nuanced responses point to its ability to enhance student learning. Further, many researchers expect that academia will adapt its teaching and assessment practices to accommodate the new reality of living, working, and learning in a world where AI is freely available [15].

Nevertheless, these general higher education policies cannot be applied directly to medical education. This is because medical education is not only a type of higher education but also a place to acquire the professional competencies required for postgraduate work. Hence, as AI is being used routinely in clinical and medical research workplaces, literacy of information technologies, including AI and GAI, should be part of competencies acquired at graduation. Moreover, there is a need to focus on teaching students how to use GAI and similar tools in an ethical way that fosters critical thinking [16].

However, strategies for introducing GAI into medical education curricula that consider the unique characteristics of medical education have not yet been established. Medical education curricula should be blended sophisticatedly based on educational strategies, assessment, the educational environment, and the individual students’ learning style [17]. In this context, GAI, especially in competency-based education, which is the standard curricular concept in medical education today, aims to help students acquire the competencies they should be able to demonstrate after graduation. Furthermore, while faculty usually take the lead in curriculum development, it is worthwhile to incorporate the views of medical students—the medical professionals of the near future.

Although experts have provided general reviews on the implementation of GAI in medical education curricula [1,3,18], there have been no reports discussing the problems and challenges in the actual process of adaptation in medical schools. Therefore, in response to the call for discussing the challenges of the adaptation process, this study analyzes the impact of GAI on medical education curricula and strategies in the context of faculty development. This is a critical time in the history of medical education that requires a new paradigm, and this study intends to add value through collaborations between educators and students in the context of ongoing innovation.

We pose the following research questions: (1) How does GAI affect curricula in undergraduate medical education? and (2) How should the medical school curriculum be reformed to address the impact of GAI?

## Methods

### Context

This study was conducted at Chiba University School of Medicine, a national university in Japan. All medical schools in Japan offer a 6-year curriculum to students entering after high school [19] and share a model core curriculum (MCC) as the nationally uniform exit competency for certifying medical degrees. The latest MCC (revised in 2022) lists the ability to recognize and use information technology, including AI, as 1 of the 10 core competencies; the achievement objectives included in the MCC define approximately two-thirds of each university’s curriculum, with the remainder allowed to be unique to each university [20].

As part of our official faculty development program, we organized a workshop for faculty and students to collaborate and invited participants from both faculty and students in August 2023. A detailed lecture on basic theory and general functions of GAI by an AI expert (EK) was conducted just prior to the workshop. Then, the participants were divided into groups of 7-8 faculty members and students to answer the above research questions. We invited faculty participants from all of the 58 departments in our school, and 47 departments agreed. Student participants were selected from students who had attended formal meetings. No participants but 1 had any experience with credited courses on AI or GAI.

### Strength, Weakness, Opportunity, and Threat (SWOT) Framework

We used the strength, weakness, opportunity, and threat (SWOT) framework for the workshop (Table 1) [20-23]. In analyses using the SWOT framework, the implementer identifies 4 internal or external components of stakeholders. Strengths refer to internal elements that facilitate the achievement of goals, whereas weaknesses refer to internal elements that hinder the achievement. Opportunities are external aspects that help stakeholders achieve their goals, including both positive environmental aspects and opportunities to initiate new activities. Threats are external aspects that can obstruct achieving goals [24]. The SWOT framework was first described academically by Learned et al [25] and has been used as an important tool for dealing with complex strategic situations by reducing the amount of information to improve decision-making. Specifically, it has been used to find gaps and matches between competencies and resources and the business environment [26] because it can assess alternatives and complex decision-making situations. In particular, education and health care are both major areas where SWOT has been frequently used [27]. Several examples of implementation in the medical education domain have been reported in academic journals and used for strategic planning in chaotic situations such as the COVID-19 pandemic in 2019 [28].

Following the analysis with the SWOT framework, this group work used the cross-SWOT (or TOWS [threat, opportunity, weakness, and strength]) matrix method to develop strategies (Table 2) [28,29]. Cross-SWOT analysis combines the relationships between internal and external environmental factors resulting from the SWOT analysis in a 2 × 2 grid to devise strategies for each of the 4 categories (strength and opportunity [SO], weakness and opportunity [WO], strength and threat [ST], and weakness and threat [WT]). The SO category focuses about how to exploit strengths for maximizing the potential opportunities. The ST category examines how threats can be transformed to opportunities. The WO category considers how to overcome the weaknesses with the opportunities. The WT category highlights how to avoid threats by minimizing weaknesses.

We chose the SWOT and cross-SWOT methods for this study because, first, they are suitable for our research questions since brainstorming questions can be used to reach a consensus. Further, these methods can establish strategies based on external as well as internal factors and barriers as well as facilitators. This feature is crucial for medical education, whose connection with society cannot be ignored.

All group work was tabulated and recorded on Google spreadsheets. To promote common discussions and minimize differences between groups, each group was assisted in discussions and work by 1 facilitator trained in SWOT analysis, in addition to the participants.

**Table 1:** The strength, weakness, opportunity, and threat (SWOT) framework.

Environmental factor	Positive effect	Negative effect
Internal	Strengths (S)	Weaknesses (W)
External	Opportunities (O)	Threats (T)

**Table 2:** The cross–strength, weakness, opportunity, and threat (SWOT) matrix.

	Strengths (S)	Weaknesses (W)
Opportunities (O)	SO	WO
Threats (T)	ST	WT

### Analysis

Qualitative content analysis was conducted to analyze the comments in the product of group work consisting of SWOT and cross-SWOT [30]. The analysis comprised the descriptions of the manifested content and interpretations of latent content [31]. Further, 4 researchers (IS, HK, KS, and NA) read the comments at the discussion and coded them to identify themes that emerged from the qualitative data independently, followed by SI checking the analysis. Points of disagreement on the data were discussed by all authors, and consensus was reached.

### Ethical Considerations

This study was performed following the Declaration of Helsinki and approved by the ethics committee or institutional review board at Chiba University Graduate School of Medicine (3425). All participants were informed in advance via a written document that their opinions would be recorded anonymously and analyzed collectively and that they could not withdraw after participation. The participants then communicated their consent on paper.

## Results

A total of 55 participants (49 faculty and 6 students) discussed the group work, and all agreed to participate in this study. Table 3 shows the specialty of faculty (basic, clinical, or social science), affiliation, and gender of the participants. Students were assigned to all groups.

In terms of the impact of GAI, content analysis of the discussions using the SWOT framework resulted in 169 items, from which 5 themes were established (improvement of teaching and learning, improved access to information, inhibition of the existing learning processes, problems in GAI, and changes in physicians’ professionality). These themes were categorized into positive, negative, and both positive and negative impacts based on the bias of the SWOT analysis (Table 4).

**Table 3:** Overview of the participants.

Characteristics				Value, n (%)
**Faculty (n=49)**				
	**Specialty**			
		Clinical sciences	25 (51)	
		Basic sciences	20 (41)	
		Social sciences	4 (8)	
	**Title**			
		Professor	8 (16)	
		Associate professor	11 (22)	
		Senior lecturer	18 (37)	
		Assistant professor	11 (22)	
		Other faculty	1 (2)	
	**Gender**			
		Men	44 (90)	
		Women	5 (10)	
Medical students (n=6)				
	**Gender**			
		Men	5 (83)	
		Women	1 (17)	

**Table 4:** Impact of GAIs^a^ in medical education curriculum.

Themes and subthemes				Items, n		Strengths, n		Opportunities, n	Weaknesses, n	Threats, n
**Positive**										
	**Improvement of teaching and learning**			48		37		11	0	0
		Assistance in creating and innovation	15		—^b^		—		—	—
		Assistance in preparing documents and other materials	12		—		—		—	—
		Improved efficiency of educational work	9		—		—		—	—
		Easier generation of virtual cases	7		—		—		—	—
		Improved efficiency of learning	5		—		—		—	—
	**Improvement of information access**			20		14		6	0	0
		Easier information gathering for students	15		—		—		—	—
		Improved literacy of second languages	5		—		—		—	—
**Negative**										
	**Inhibition of the existing learning processes**			36		0		0	29	7
		Decreased ability to think on their own	24		—		—		—	—
		Inhibition of the existing assessment methods	8		—		—		—	—
		Superficial academic learning in cognitive areas	4		—		—		—	—
	**Potential problems in GAI**			28		0		0	11	17
		Doubt of authenticity	19		—		—		—	—
		Ethical issues	7		—		—		—	—
		Information leakage	2		—		—		—	—
**Both positive and negative**										
	**Changes in physicians’ professionality**			37		7		13	4	13
		Declining value of the knowledge of expertise	11		—		—		—	—
		Revising up the value of face-to-face encounters	10		—		—		—	—
		Volatile roles of physicians in the future	6		—		—		—	—
		Increased efficiency of clinical and research work	6		—		—		—	—
		Improved ability of patients to gather medical information	4		—		—		—	—

^a^GAI: generative artificial intelligence.

^b^Not applicable.

In total, 1 positive impact was the improvement of teaching and learning. The faculty members believed that GAI would assist them in creating better instructional content and materials and help them become more efficient. Students also thought that GAI could be incorporated to assist them in the learning process, for example, summarizing information. Further, GAI could be useful as a new emergent tool because it allows students to suggest ideas. The participants also noted that clinical education requires resources such as case scenarios and images, and the ability to generate them would be useful for education. Representative comments are presented below (note that the symbols in parentheses indicate the identification number of each comment; S, W, O, and T denote the SWOT matrix categories that were described in Table 1).

Faculty can use GAI to produce quality resumes.

S601

GAI saves time in making slides for lectures.

S101

Students can pick up the key points they learn.

S408

Another positive impact was improved access to information: the participants believed that the GAI would make it easier for students to gather information, given that the use of GAI is simpler than traditional search functions. Further, in cultures where English is not the native language, such as Japan, literacy in English—the de facto standard academic language of the world—is a major issue. Hence, participants expected that facilitating literacy in second languages, such as English for Japanese students, would improve the curriculum.

The GAI response could be used to obtain opinions on various aspects.

S402

GAI can translate English literature easiliy.

O501

GAI can save time in searching for new information.

S605

Conversely, 2 themes were identified as negative impacts. First, teachers were concerned that the use of GAI would reduce learners’ ability to think independently. It was also noted that existing learner assessment methods would be less applicable and that continuing with existing learning strategies would result in lower orders in the cognitive domain. Second, potential problems in GAI such as doubt of authenticity and ethical issues were concerned.

Some students may finish learning only by memorizing superficial knowledge.

W104

Students will have less opportunity to think for themselves.

W601

If students write reports and essays with the assistance of GAI, it would be impossible to assess them properly.

T106

We are not sure if the information output by GAI is really correct. We need to accept the assumption that it may contain incorrect information.

T101

Copyright and portrait rights issues have not been resolved.

T201

Further, a third major, both positive and negative, impact was identified as the change in physicians’ professionality. Subthemes included those related to the positioning of medical expertise, such as the declining value of specialized knowledge and the ability to gather information from patients, and those that were summative, such as the changing nature of work due to labor-saving clinical and research work, the resulting reevaluation of face-to-face contact, and the increased volatility of the roles of future physicians.

Patients and family members can use GAI to obtain medical knowledge.

T304

Physicians will no longer be expected to just know expert knowledge.

T801

The competency to interact directly with patients and their families will be more important.

T108

The paperwork burden of writing medical certificates and charts will be reduced.

O702

Errors in some of clinical routine work can be reduced by replacing the physicians.

O703

Creative work will be left to humans.

T203

In terms of medical education curriculum reform strategies to address the impact of GAI, content analysis of results of the cross-SWOT analysis established 3 themes from 104 items (learning about GAI, learning with GAI, and learning aside from GAI; Table 5).

**Table 5:** Strategies of curriculum reform to address the impact of GAI^a^.

Themes and subthemes		Items, n	SO^b^, n	ST^c^, n	WO^d^, n	WT^e^, n
**Learning about GAI**		22	0	2	7	13
	Characteristics of GAI	14	—^f^	—	—	—
	Appropriate use of GAI in medicine	4	—	—	—	—
	Ethics and compliance	4	—	—	—	—
**Learning with GAI**		57	35	12	8	2
	Improvement of learning efficiency	21	—	—	—	—
	Generating educational materials	16	—	—	—	—
	Support for information gathering and dissemination	10	—	—	—	—
	Adaptive learning	4	—	—	—	—
	Support for group learning	3	—	—	—	—
	Promoting case-based learning	3	—	—	—	—
**Learning aside from GAI**		25	3	8	5	9
	Maintaining the GAI-free learning process	8	—	—	—	—
	Fostering higher cognitive domain of learning	8	—	—	—	—
	More communication exercises	6	—	—	—	—
	Participation and experience in the workplace	3	—	—	—	—

^a^GAI: generative artificial intelligence.

^b^SO: strength and opportunity.

^c^ST: strength and threat.

^d^WO: weakness and opportunity.

^e^WT: weakness and threat.

^f^Not applicable.

As for learning about GAI, participants suggested that in addition to learning the characteristics of GAI, they should learn about the proper use of GAI in medicine as well as ethics and compliance to mitigate its impact. These topics were suggested primarily in response to the weakness of the medical education curriculum. Representative comments are presented below (note that the symbols in parentheses indicate the identification number of each comment; SO, WO, ST, and WT denote the cross-SWOT matrix categories as described in Table 2).

Understanding pitfalls of GAI.

WT504

Learning how to use GAI in the clinical and learning process.

WO603

Implementing information ethics education.

WO404

Learning with GAI was proposed for the use of GAI in existing curricula. Subthemes were obtained to improve the efficiency of learning and to support to gather and disseminate information. Further, it was suggested that teachers could also use GAI to generate educational materials. Moreover, participants pointed out that supporting adaptive and group learning as well as promoting the use of digital patients who are more diverse than real patients into education would introduce clinical case-based learning.

Summarizing the outline of the learning content with GAI.

WO201

Creating self-assessment drills to support learning.

SO201

Providing learning content based on career plans and level of understanding.

SO503

Utilizing GAI to support learning achievement for each small group.

WO801

Providing more clinical encounters with virtual patients.

SO305

Finally, learning that does not rely on GAI was also identified as one of the subjects to focus on in medical education curricula. As subthemes, the participants suggested maintaining the GAI-independent learning process, introducing higher order learning into the learning of knowledge domains, providing more communication exercises, and promoting participation and experience in the workplace, such as clinical clerkships.

Placing more emphasis on performance assessment than on essay or writing tests.

WT303

Promoting interactive learning.

WO502

Improving humanistic professional skills, such as empathy for patient concerns.

ST702

Reducing lectures and increasing skills training and clinical clerkships.

SO402

## Discussion

### Principal Findings

In this study, the impact of GAI on medical education curricula and the direction of curriculum reform based on the existence of GAI were investigated in the context of faculty development. In medical education research, the same attempt has been made by previous studies to summarize the results of SWOT analyses through qualitative analysis [21,32], which is an appropriate approach for gathering stakeholders’ opinions on the impact of an issue. Similarly, in this study, the inclusion of students in addition to medical school faculty from all areas of medical education in this study of GAI curriculum reform helped to strengthen the conclusions about GAI.

In recent years, the use of GAI in medical education has been the subject of only a few recommendations by some experts and reports of advanced practices [33,34]. However, the general state of medical education curricula has not yet been well defined, and to our knowledge, no study has yet investigated and summarized the needs of medical schools and their faculty members who manage the actual curriculum. Among the study’s findings, the need to learn about the shortcomings of GAI and some of the specific ideas for incorporating GAI into existing educational strategies were consistent with the recommendations of experts [34]. For example, Boscardin et al [33] have compiled a resource for medical educators to increase their AI literacy.

Further, the application of GAI may be particularly effective for learning in disciplines such as clinical medicine [35], where decisions are based on background knowledge backed by solid evidence. In clinical reasoning [36], for example, GAI can extend our views on a problem. Ultimately, it can add several differential diagnoses that we may not have thought of. Hence, the combined use of GAI in the clinical workplace is expected to be part of a physician’s skill set and, through training, will be expected to assist physicians in the practice [33].

Interestingly, we found a new approach that can promote the use of simulated patients in education and adaptive learning as part of “learning with GAI.” The educational use of digital patients can complement learners’ clinical experiences through experiential learning theory, providing a mechanism for information gathering and clinical decision-making in a safe environment [37]. While generally useful for understanding standardized clinical conditions [38], participants expected that the educational significance of digital patients could be amplified through the use of GAI, which can easily generate a wide variety of problems. Digital patients with GAI can even be expected to acquire some interactivity [39].

The ability to use GAI to create a variety of educational resources with less effort is also a key factor in promoting adaptive learning [40]. It is hoped that the incorporation of good practices into GAI-made educational resources will lead to the practical application of these appropriate strategies in medical education curricula. As a letter indicates, “learning with GAI” may apply to “learning about GAI” as well [41].

Furthermore, we found perspectives in this report that have not been recommended by AI experts in the past. A distinctive example is the recommendation that curricula should focus on “changes in physicians’ competencies” as the impact of GAI and “learning aside from GAI” to respond to these impacts. In areas such as medicine, where competencies of health professions are indispensable human resources, competencies that cannot be replaced by GAI, as listed from the cross-SWOT analysis in our study, will continue to be required, and as GAI becomes more prevalent in the clinical workplace, the role of physicians is expected to be focused on tasks that cannot be replaced by AI. For example, experience in the workplace cannot be replaced by GAI, nor can evaluation be faked, and hence will be emphasized more in future medical education curricula. Moreover, learners could expect to reach higher orders of cognitive domain such as “apply” and “analyze,” rather than “know” and “understand.” In this respect, evaluating GAI-generated information could also be an effective learning and assessing approach.

In terms of learners’ assessment, participants were concerned that existing assessments using students’ output may be less reliable. This suggests that summative assessments using high-stakes testing, at least in the knowledge domain, will be harder to implement. Instead, a novel assessment system has been proposed, by which the utility of assessment can complement the former approach by integrating the results of various types of feedback opportunities programmatically [42]. Such a concept of assessment may become increasingly important in future medical education curricula. Simultaneously, the importance of assessing skills and attitudes will increase because they will account for a greater proportion of a physician’s competence. These transitions are consistent with, and in fact promote, the paradigm shift in medical education, noted a decade ago [43].

Another interesting aspect of our report is that faculty and students proposed their own strategies for curriculum reform based on the impact of GAI through faculty development. Our attempt enabled faculty to engage their own intentions in the university’s curriculum reform. Classical faculty development has often adopted a top-down approach for communicating educational know-how and policies [44]. Similarly, policies for the use of GAI that have been formulated in various countries and several universities have adopted the same top-down approach. However, in curriculum reform, the usefulness of a bottom-up approach that takes advantage of faculty members’ initiative has long been pointed out [45]. Specifically, adopting a bottom-up approach facilitates faculty consensus on the curriculum and minimizes gaps in the objectives of reform and what needs to be done. In this respect, GAI is not just an educational device or technique; rather, it has the potential to revolutionize education, and its technological progress is rapid. When such an innovative technology is quickly introduced, it is essential that the entire organization is ready to embrace change [46]. If faculty development with respect to GAI is conducted by teaching expert knowledge and providing recommendations on how to (or not to) use GAI, the faculty development will have a limited effect for curriculum reform. Conversely, our faculty development program allows faculty members to formulate their own curriculum reform proposals and thus is expected to lead to the introduction of more effective ways of incorporating GAI in the context of each university. Among the items mentioned in the results of analysis, the negative impacts of GAI and the reform strategies to overcome them had much in common with public proposals, but it is significant that the faculty members themselves were able to outline their own proposals. Furthermore, Steinert et al [46] points out that faculty development is also a place of community of practice. From this perspective, it is significant that students, who are stakeholders in the learning process, are also involved, and the fact that the GAI has provided an opportunity to incorporate students’ opinions on matters that will have a large impact on their future as health professionals will give validity to the reformed curriculum. The strategies we developed were incorporated into the *Guidelines on the Use of Generative AI in Teaching and Learning* in our university in October 2023 (an internal document). We believe that this adoption suggests that the findings of our efforts are highly useful.

### Limitations

This study had several limitations. First, it was conducted at a single medical school in Japan. Since medicine is highly context dependent, future implementation of GAI in medicine may vary greatly depending on cultural and curricular characteristics. However, certain commonalities in higher education and undergraduate medical education competencies may serve as an example of how GAI may be used. Second, the number of students was smaller than that of faculty, and although the facilitator encouraged participants to pay attention to the issue of hierarchy and generate opinions during the workshop, he did not incorporate any structural devices to eliminate the issue of hierarchy between students and faculty. However, since the issue of GAI has a common impact on faculty and medical students, we do not expect that hierarchy had a significant impact on the product. Third, although the clinical workplace contains multiple health professions besides physicians, we did not incorporate the opinions of other professionals than physicians this time. In the future, similar workshops with interprofessional participants should incorporate more diverse opinions to further enhance the relevance of the developed strategies.

### Conclusions

We conducted a qualitative analysis of the impact of GAI on medical education curricula and strategies for responding to it using the SWOT framework and cross-SWOT matrix. We recruited faculty and students to identify both positive and negative impacts of GAI on medical education curricula as well as “changes in physician specialties” as a characteristic of medical education. Curricular response principles were broadly classified into “learning about GAI,” “learning with GAI,” and “learning aside from GAI.” These principles will be the 3 pillars of medical education curriculum reform in the GAI era. Particularly, it is crucial to investigate how to maintain and promote learning aside from GAI.

## Abbreviations

AI: artificial intelligence
GAI: generative artificial intelligence
LLM: large language model
MCC: model core curriculum
SO: strength and opportunity
ST: strength and threat
SWOT: strength, weakness, opportunity, and threat
TOWS: threat, opportunity, weakness, and strength
UNESCO: United Nations Educational, Scientific and Cultural Organization
WO: weakness and opportunity
WT: weakness and threat
